# Crystal structure of (*E*)-*N*-(4-bromo­phen­yl)-2-cyano-3-[3-(2-methyl­prop­yl)-1-phenyl-1*H*-pyrazol-4-yl]prop-2-enamide

**DOI:** 10.1107/S2056989024003086

**Published:** 2024-04-23

**Authors:** Mamdouh A. Abu-Zaied, Reham A. Mohamed-Ezzat, Galal H. Elgemeie, Peter G. Jones

**Affiliations:** a Green Chemistry Department, Chemical Industries Research Institute, National Research Centre, Dokki, Giza, Egypt; b Chemistry of Natural & Microbial Products Department, Pharmaceutical and Drug Industries Research Institute, National Research Centre, Cairo, Egypt; c Chemistry Department, Faculty of Science, Helwan University, Cairo, Egypt; d Institut für Anorganische und Analytische Chemie, Technische Universität Braunschweig, Hagenring 30, D-38106 Braunschweig, Germany; Tokyo University of Science, Japan

**Keywords:** pyrazole, acryl­amide, hydrogen bonds, twinning, crystal structure

## Abstract

The structure of the title compound, C_23_H_21_BrN_4_O, contains two independent mol­ecules connected by hydrogen bonds of the type N_amide_—H⋯N≡C to form a dimer. The configuration at the exocyclic C=C double bond is *E*. The mol­ecules are roughly planar except for the isopropyl groups. There are minor differences in the orientations of these groups and the phenyl rings at N1. The dimers are further linked by ‘weak’ hydrogen bonds, two each of the types H_phen­yl_⋯O=C (H⋯O = 2.50, 2.51 Å) and H_phen­yl_⋯Br (H⋯Br = 2.89, 2.91 Å), to form ribbons parallel to the *b* and *c* axes, respectively. The studied crystal was a non-merohedral twin.

## Abstract 2

The two independent mol­ecules of C_23_H_21_BrN_4_O are closely similar and roughly planar except for the isopropyl groups. They are connected by hydrogen bonds of the types N_amide_—H⋯N≡C, H_phen­yl_⋯O=C and H_phen­yl_⋯Br.

## 1. Chemical context

Pyrazoles contribute significantly to medicinal applications (Bennani *et al.*, 2020 ▸; Ansari *et al.*, 2017 ▸); their pharmaco­logical activity is reflected in their presence in various therapeutic agents (Küçükgüzel & Şenkardeş, 2015 ▸), *e.g*. as agents against human colon cancer, leukaemia and melanoma (Elgemeie & Mohamed-Ezzat, 2022 ▸), as anti-inflammatory agents, and in the field of anti­viral therapeutics against various targets such as CoX-1, CoX-2, NNRTI, HSV-1 and H1N1 (Khan *et al.*, 2016 ▸; Li *et al.*, 2015 ▸). Heterocyclic compounds containing pyrazole rings are also efficacious components in many multi-component syntheses (Tu *et al.*, 2014 ▸); we have reported and reviewed their use as novel synthetic inter­mediates (Elgemeie *et al.*, 2015 ▸; Abu-Zaied *et al.*, 2018 ▸, 2019 ▸; Metwally *et al.*, 2024 ▸). We have also synthesized various pyrazole-fused heterocyclic compounds as bioactive agents acting as anti­metabolites (Elgemeie & Abu-Zaied, 2015 ▸; Elgemeie *et al.* 2017*a* ▸,*b* ▸, 2019 ▸; Mohamed-Ezzat & Elgemeie, 2023 ▸). 
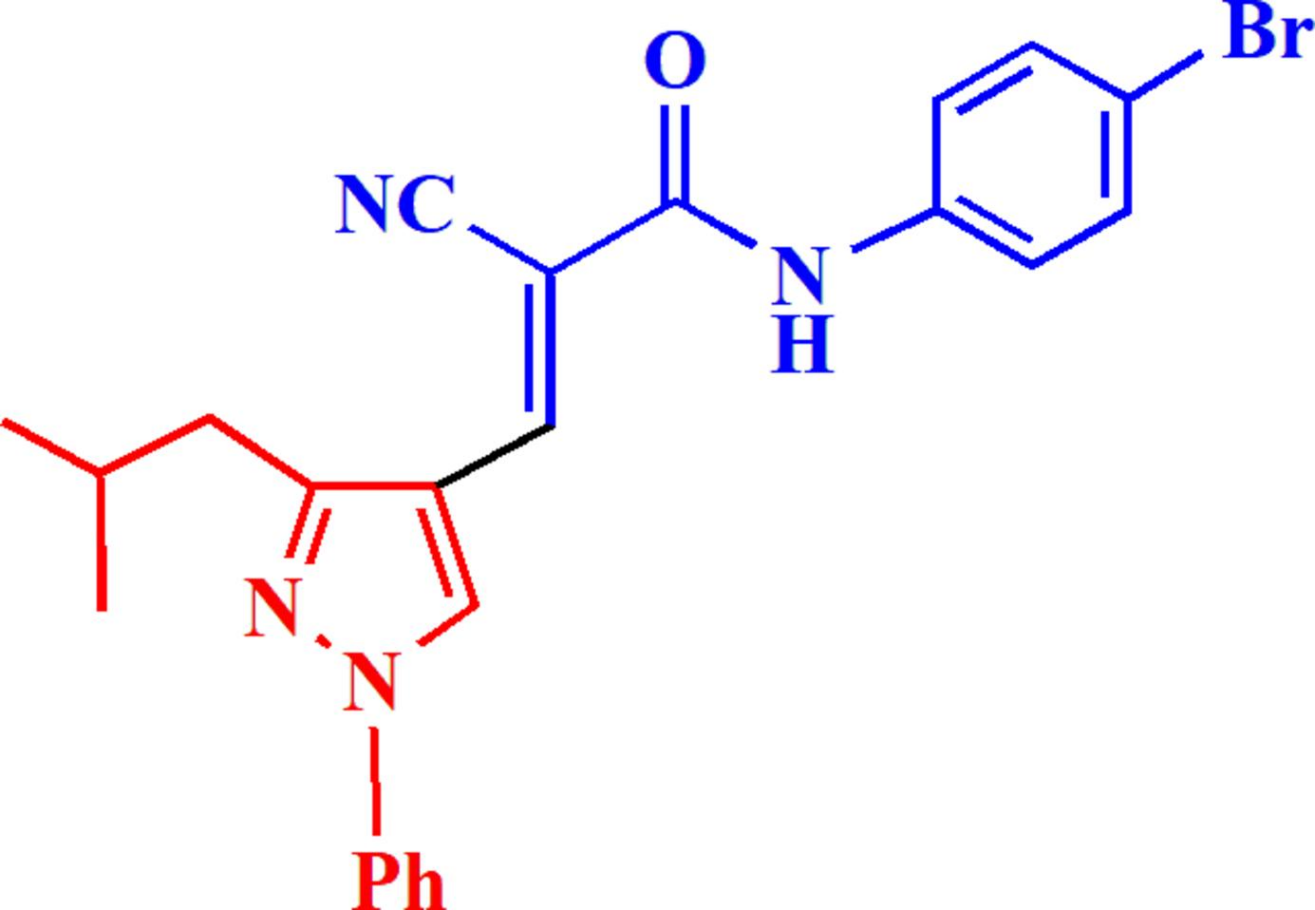


**Figure 1:**
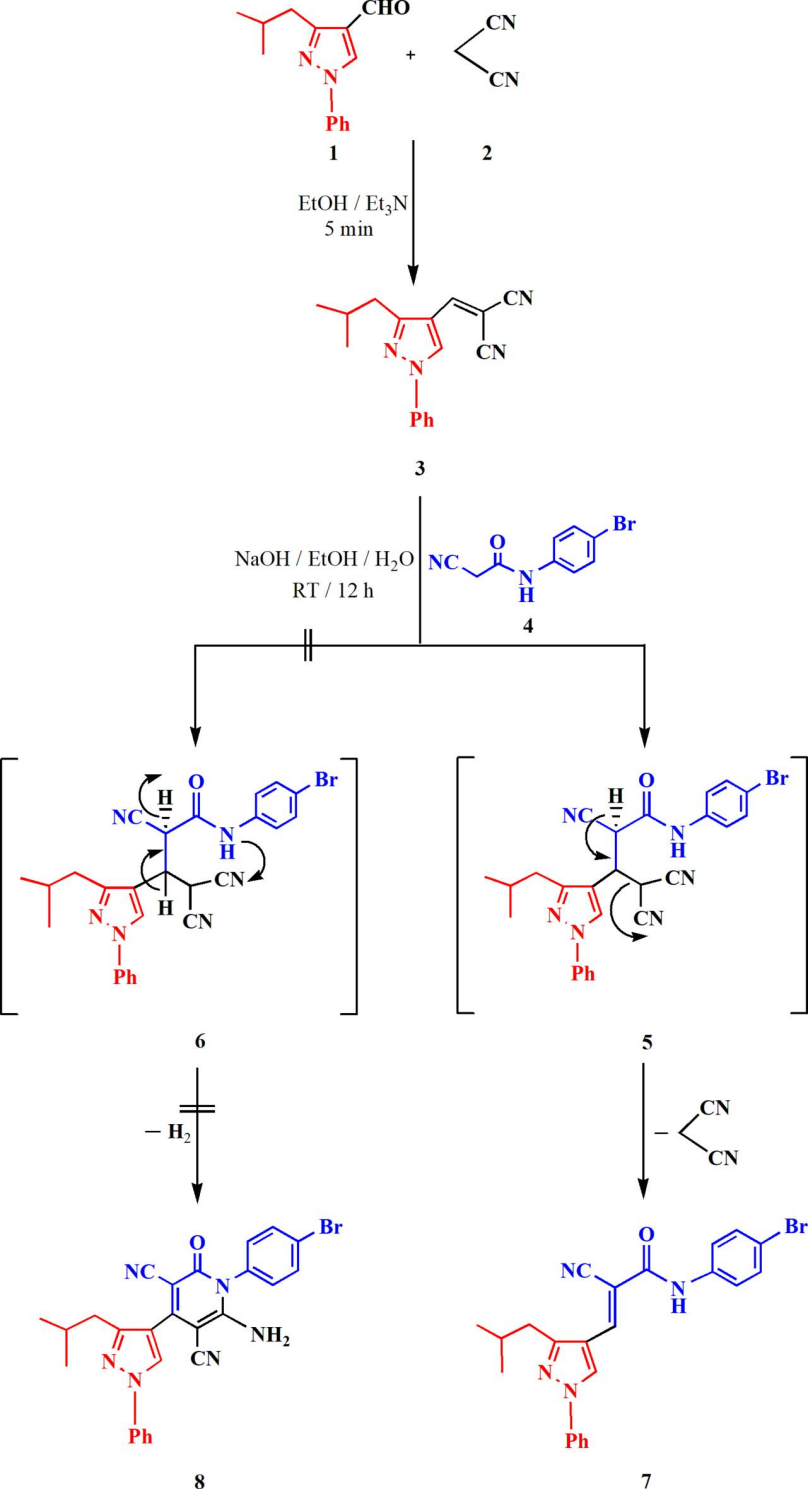
The reaction scheme leading to the title compound **7**.

In this article, we report the synthesis of the title compound **7**, which bears a substituted acryl­amide side chain at the 4-position of the pyrazole ring, by the reaction between 2-[(3-isobutyl-1-phenyl-1*H*-pyrazol-4-yl)methyl­ene]malono­nitrile **3** and *N*-(4-bromo­phen­yl)-2-cyano­acetamide **4** in an ethanol–water mixture in the presence of sodium hydroxide (see Fig. 1 ▸, where the suggested mechanism is also shown). The reaction starts by the nucleophilic attack of the active methyl­ene group of **4** at the double bond of **3** to give an inter­mediate Michael addition product **5**, which eliminates malono­nitrile to give the final product **7**. The structure of **7** was confirmed *via* spectroscopic techniques; thus, the IR spectrum indicated the presence of a characteristic NH absorption band at 3455 cm^−1^, and the ^1^H NMR spectrum revealed the presence of an NH signal at 10.36 ppm, a singlet vinylic signal at 8.10 ppm, and aromatic protons at 7.52–7.63 ppm. It is possible that compound **7** is the thermodynamically controlled product because of lower steric hindrance, and is thus formed instead of the *N*-aryl-2-pyridone **8** *via* inter­mediate **6**. The structure of compound **7** has now been been unambiguously confirmed by single-crystal X-ray diffraction and is presented here.

## 2. Structural commentary

**Figure 2:**
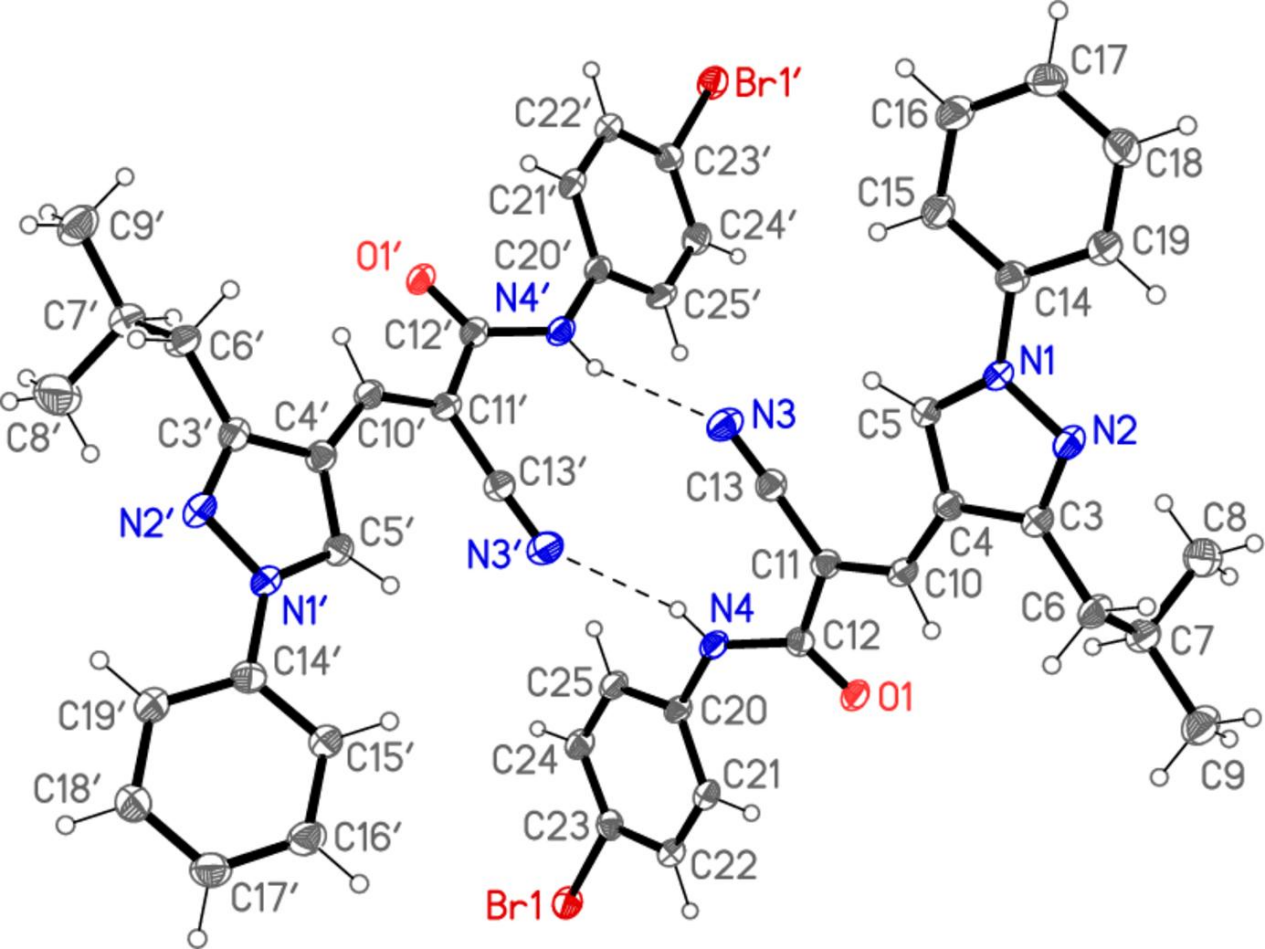
The structure of compound **7** in the crystal; two independent mol­ecules are linked by hydrogen bonds (shown as dashed lines). Ellipsoids represent 50% probability levels.

The structure of compound **7** is shown in Fig. 2 ▸, with selected mol­ecular dimensions in Table 1 ▸ (and hydrogen bonds in Table 2 ▸); there are two independent mol­ecules in the asymmetric unit. The configuration at the double bond C10=C11 is *E*, with the amide and pyrazolyl groups mutually *trans*, which leads to short intra­molecular contacts H10⋯O1 of 2.43, 2.44 Å. The two independent mol­ecules are linked to form a dimer by hydrogen bonds of the type N_amide_—H⋯N≡C; the graph set is 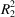 (12). The atom numbering of both mol­ecules is the same, but with the addition of primes (′) for the second mol­ecule. The centre of gravity of the asymmetric unit lies close to the point (0.5, 0.75, 0.5).

**Figure 3:**
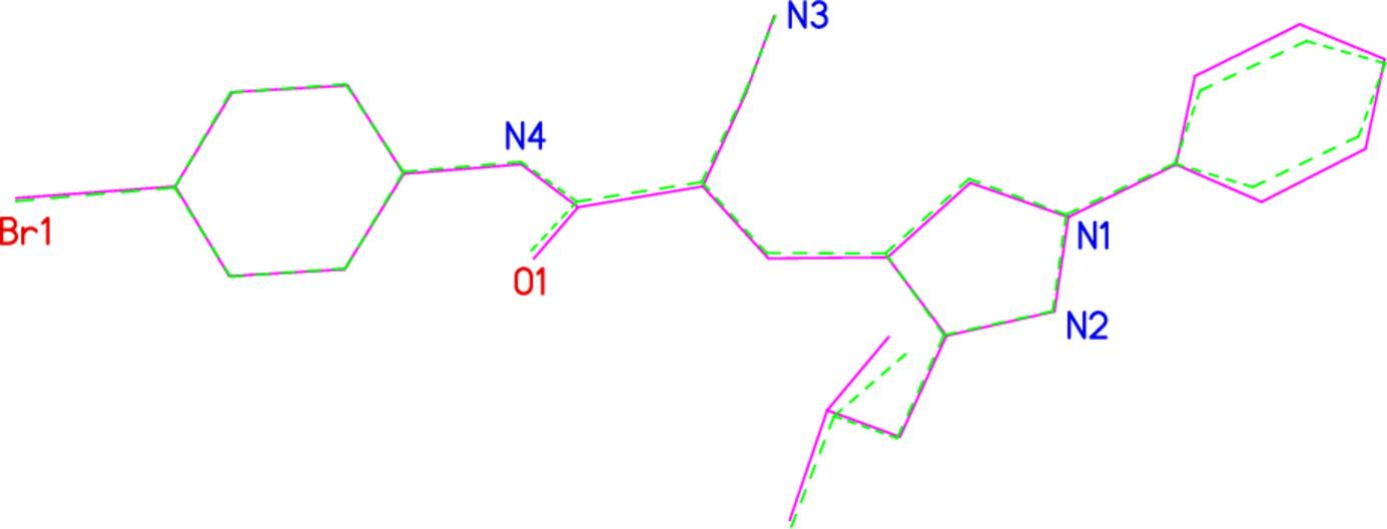
A least-squares fit of both independent mol­ecules. The second independent mol­ecule is indicated by dashed green bonds.

**Figure 4:**
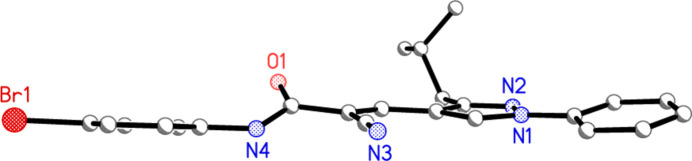
Side view of mol­ecule 1 (hydrogen atoms omitted).

Bond lengths and angles may be considered normal, although the narrow angles in the pyrazole rings are necessarily reflected in some wide exocyclic angles, and the angle C4—C10—C11 is also wide. A least-squares fit of both mol­ecules (for all atoms except hydrogens; Fig. 3 ▸) gave an r.m.s. deviation of 0.12 Å, with minor differences in the orientations of the isopropyl groups and the ring C14–19 (the latter involving torsion angle differences of *ca* 13°). This corresponds to the presence of a local twofold axis passing through the centre of gravity of the asymmetric unit. A side view of mol­ecule 1 shows that it is very roughly planar except for the isopropyl group (Fig. 4 ▸). The inter­planar angles between the pyrazole ring and rings C14–C19 and C20–C25, respectively, are 10.4 (2), 22.5 (2)° in mol­ecule 1 and 10.3 (2), 8.9 (2)° in mol­ecule 2. Another factor associated with the lack of planarity is the central torsion angle of the atom sequence C4—C10—C11—C12—N4—C20, which differs by *ca* 24° from the 180° required for an ideally extended sequence. The geometry at the amide nitro­gen atoms is almost exactly planar (r.m.s. deviations from the best plane through the nitro­gen and its immediate substituents are 0.012 and 0.004 Å for the two mol­ecules).

## 3. Supra­molecular features

**Figure 5:**
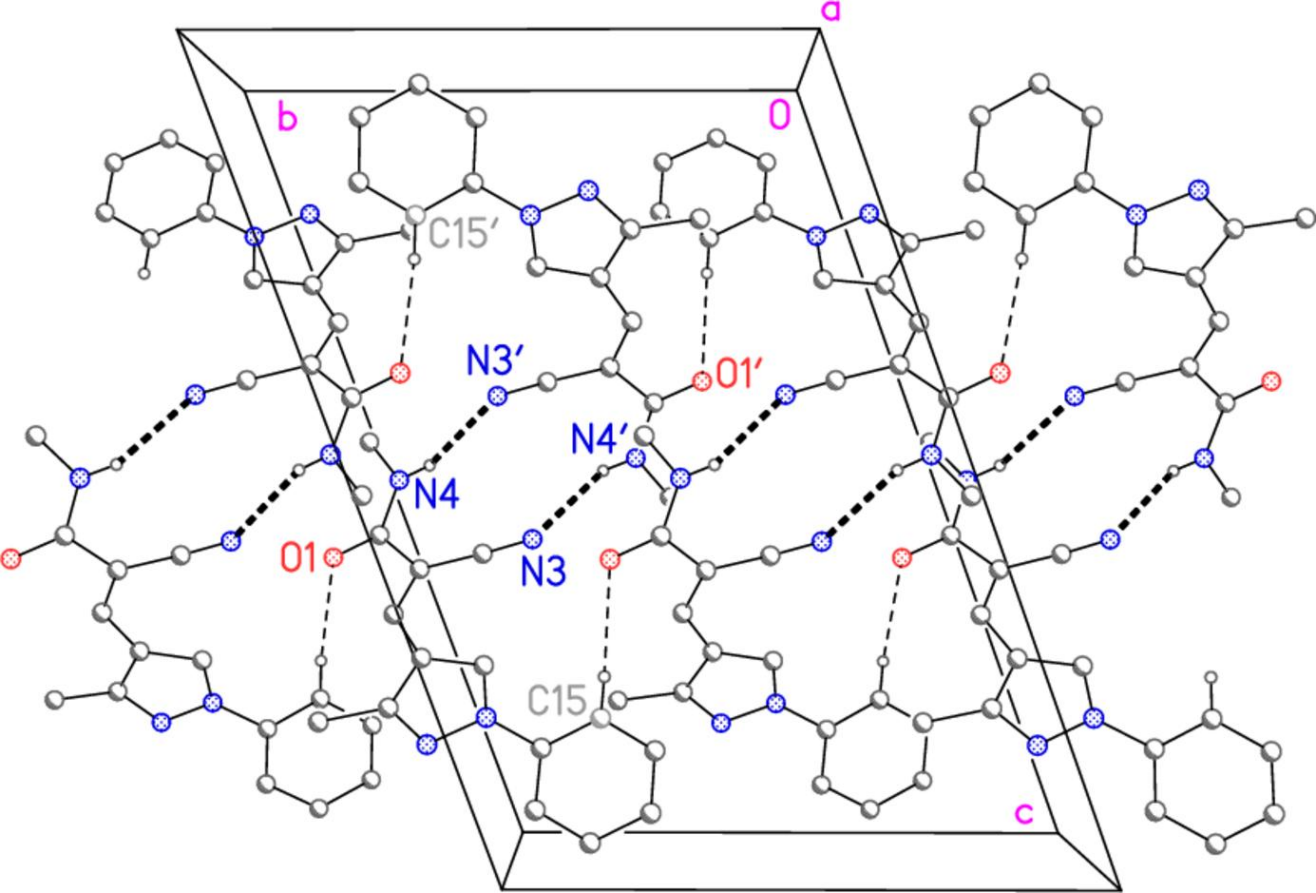
Packing diagram of compound **7** viewed parallel to the *a* axis. The dashed bonds indicate classical (thick) or ‘weak’ (thin) hydrogen bonds. For clarity, the bromo­phenyl rings have been reduced to their *ipso* carbon atoms and the isopropyl groups are omitted, as are the hydrogen atoms not involved in hydrogen bonding.

**Figure 6:**
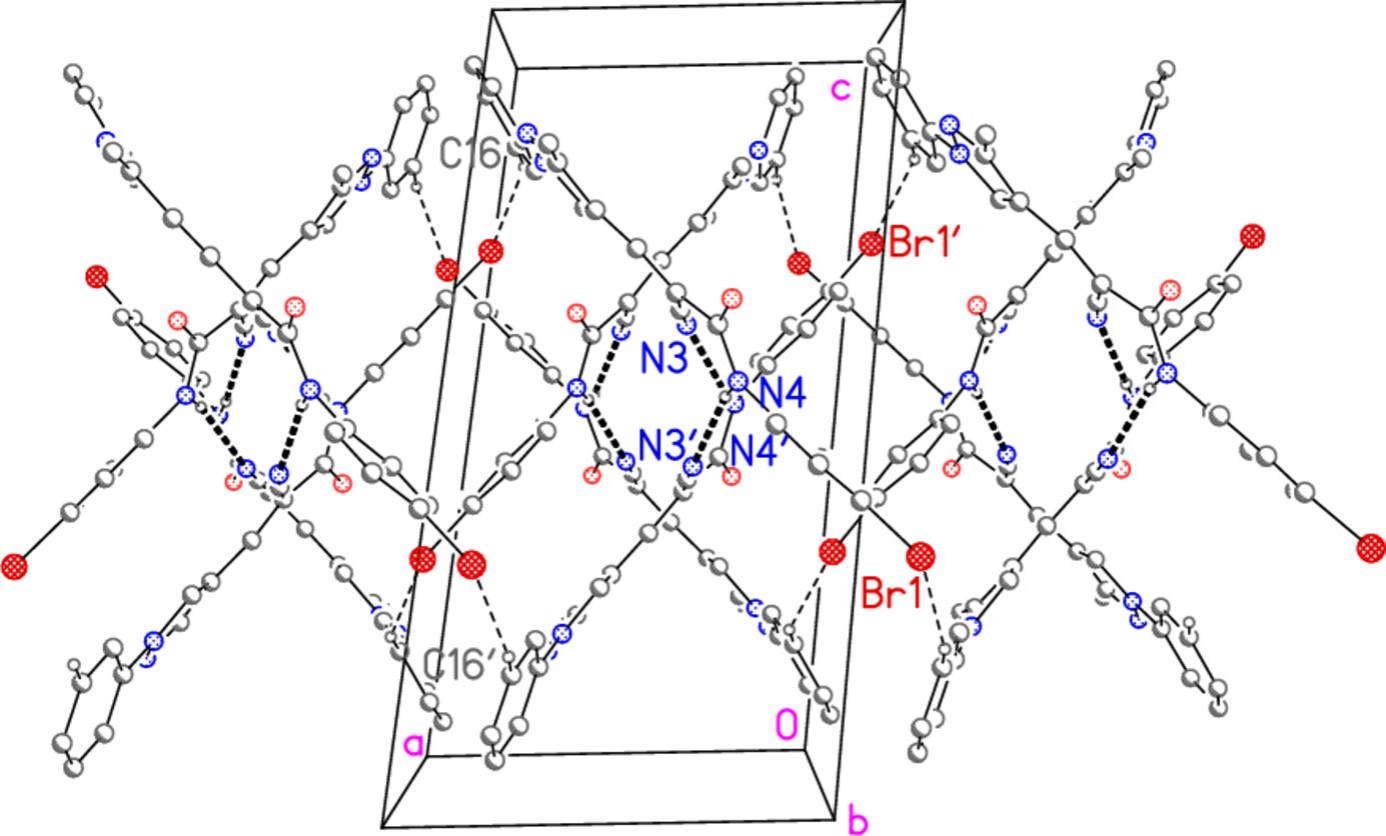
Packing diagram of compound **7** viewed parallel to the *b* axis. The dashed bonds indicate classical (thick) or ‘weak’ (thin) hydrogen bonds. For clarity, the isopropyl groups are omitted, as are the hydrogen atoms not involved in hydrogen bonding.

The association of the two independent mol­ecules to form a hydrogen-bonded dimer was discussed in the previous section. The common hydrogen-bonding pattern for amides, with dimer formation *via* two N—H⋯O=C bonds, is not observed; this would require rotation around the amide C4—N12 bond to make the sequence O1=C12—N4—H4 synperiplanar rather than anti­periplanar, which would presumably involve a close approach of the bromo­phenyl and nitrile groups. There are also two pairs of ‘weak’ hydrogen bonds, namely H15⋯O1′/H15′⋯O1 and H16⋯Br1′/H16′⋯Br1 (for details see Table 2 ▸). The former link the dimers to form a ribbon structure parallel to the *b* axis (Fig. 5 ▸), whereas the latter are associated with a ribbon structure parallel to the *c* axis (Fig. 6 ▸). The combination of the two ribbons leads to the final three-dimensional packing.

## 4. Database survey

The search employed the routine ConQuest (Bruno *et al.*, 2002 ▸), part of Version 2023.3.0 of the Cambridge Database (Groom *et al.*, 2016 ▸), and sought structures with pyrazole rings substituted at C4 by the group —C=C(CN)—C(=O)—N. Only one exact hit was registered: 2-cyano-3-(1-phenyl-3-(thio­phen-2-yl)-1*H*-pyrazol-4-yl)prop-2-enamide (YEJVES; Kariuki *et al.*, 2022 ▸), which has a thio­phenyl group instead of the isobutyl group in **7**, and an unsubstituted amide group. It too shows the *E* configuration; the hydrogen bonding involves hydrogen-bonded dimers *via* N—H⋯O=C contacts between the two independent mol­ecules, crosslinked by further hydrogen bonds N—H⋯N≡C. There were, however, four other hits in which the substituent was involved in another ring fused with the pyrazole, *e.g.* methyl 5-cyano-6-oxo-3-[4-(tri­fluoro­meth­yl)phen­yl]-6,7-di­hydro-2*H*-pyrazolo­[3,4-*b*]pyridine-4-carboxyl­ate (HUVXED; Ali *et al.*, 2013 ▸).

## 5. Synthesis and crystallization

Synthesis of compound **3**

To a solution of the pyrazole-4-carbaldehyde derivative **1** (10 mmol) in absolute ethanol (10 mL) containing 3 drops of tri­ethyl­amine, malono­nitrile **2** (10 mmol) was added, and the mixture was stirred for 5 min. The precipitate thus formed was filtered off and recrystallized from ethanol to afford compound **3** as a colourless solid in 90% yield. M.p. 442 K; IR (KBr, cm^−1^) ν 3132 (C—H aromatic), 2950 (CH), 2216, 2210, (2 CN), 1612 (C=N), 1594 (C=C); ^1^H NMR (500 MHz, DMSO-*d*_6_): δ 0.90 (*d*, *J* = 6.4 Hz, 6H, 2 × CH_3_), 1.94–1.96 (*m*, 1H, CH), 2.65 (*d*, *J* = 7.2 Hz, CH_2_), 7.45–7.84 (*m*, 5H, C_6_H_5_), 8.08 (*s*, 1H, vinylic H), 9.01 (*s*, 1H, pyrazole H-5). Analysis calculated for C_17_H_16_N_4_ (276.34): C 73.89, H 5.84, N 20.27. Found: C 73.80, H 5.75, N 20.18%.

Synthesis of compound **7**

A solution of 2-[(3-isobutyl-1-phenyl-1*H*-pyrazol-4-yl)methyl­ene]malono­nitrile **3** (10 mmol) in an ethanol–water mixture (1:1) containing sodium hydroxide (10 mmol) was treated with *N*-(4-bromo­phen­yl)-2-cyano­acetamide **4** (10 mmol) and heated under reflux for 12 h. The reaction mixture was then cooled to ambient temperature, and the precipitate thus formed was collected by filtration, dried and recrystallized from DMSO to furnish compound **7** as colourless crystals in 95% yield. M.p. 497–499 K; IR (KBr, cm^−1^) ν 3455 (NH), 3045 (C—H aromatic), 2960 (CH), 2210 (CN), 1663 (C=O), 1602 (C=N), 1591 (C=C); ^1^H NMR (500 MHz, DMSO-d_6_): δ 0.93 (*d*, 6H, *J* = 6.7 Hz, 2 × CH_3_), 1.99–2.02 (*m*, 1H, CH), 2.71 (*d*, 2H, *J* = 7.15 Hz, CH_2_), 7.52–7.63 (*m*, 9H, C_6_H_5_, C_6_H_4_), 8.10 (*s*, 1H, vinylic-H), 9.00 (*s*, 1H, pyrazole H-5), 10.36 (*br*, *s*, D_2_O exch., 1H, NH); ^13^C NMR (125 MHz, DMSO-*d*_6_): δ 22.84 (2C, 2 × CH_3_), 28.91 (CH), 34.3 (CH_2_), 103 (pyrazole C4), 115.9 (C=CH), 117.45 (CN), 119.82 (2C, Ar-C), 123.41 (2C, Ar-C), 128.15 (Ar-C), 128.74 (pyrazole-C5), 130.36 (2C, Ar-C), 132.05 (2C, Ar-C), 138.22 (2C, Ar-C), 142.15 (Ar-C), 156.28 (pyrazole-C-3), 159.112 (–CH=C), 161.01 (C=O). Analysis calculated for C_23_H_21_BrN_4_O (449.34): C 61.48, H 4.71, Br 17.78, N 12.47. Found: C 61.38, H 4.60, Br 17.68, N 12.38%.

## 6. Data collection and reduction

Most crystals were fine needles that diffracted very weakly. However, a few broader laths were found, one of which was used for the data collection despite its somewhat diffuse reflection form. The reflections found in the initial cell determination were 93% indexed using a *C*-centred monoclinic cell with approximate cell constants *a* = 35.16, *b* = 12.77, *c* = 9.22 Å, β = 97.3°. During the course of the data collection, this was automatically changed to the final triclinic cell, presumably on the basis of a prohibitively high *R*_int_ value for the monoclinic cell. A closer inspection of the complete data then revealed the twinning, and the data reduction was repeated accordingly.

## 7. Refinement

The structure was refined using the ‘HKLF 5’ command as a two-component non-merohedral twin (by 180° rotation around c*), whereby the relative volume of the smaller component refined to 0.2500 (8). The two largest peaks in the residual electron density (*ca* 1.4 e Å^−3^) are arithmetically related to the coordinates of the two bromine atoms and are probably attributable to residual twinning effects. As is often the case for non-merohedral twins, some intensities were badly in error; six such reflections were omitted from the refinement. Because of the special methods involved in the data reduction of non-merohedral twins, equivalent reflections were merged and *R*(int) is thus meaningless; because reflections from both twinning components are included, the number of reflections should be inter­preted with caution. The weighting parameters did not converge, but oscillated over a small range (*e.g.* the *SHELXL* ‘a’ parameter between 0.0504 and 0.0507); arbitrarily, we chose the former value. Crystal data, data collection and structure refinement details are summarized in Table 3 ▸.

The hydrogen atoms of the NH groups were refined freely, but with the N—H distances restrained to be approximately equal (command ‘SADI’). The methyl groups were included as idealized rigid groups allowed to rotate but not tip (command ‘AFIX 137’), with C—H = 0.99 Å and H—C—H = 109.5°. Other hydrogen atoms were included using a riding model starting from calculated positions (C—H_methyl­ene_ = 0.99, C—H_methine_ = 1.00, C—H_arom_ = 0.95 Å). The *U*(H) values were fixed at 1.5 × *U*_eq_ of the parent carbon atoms for the methyl group and 1.2 × *U*_eq_ for other hydrogens.

## Supplementary Material

Crystal structure: contains datablock(s) I, global. DOI: 10.1107/S2056989024003086/jp2004sup1.cif

Structure factors: contains datablock(s) I. DOI: 10.1107/S2056989024003086/jp2004Isup2.hkl

Supporting information file. DOI: 10.1107/S2056989024003086/jp2004Isup3.cml

CCDC reference: 2347502

Additional supporting information:  crystallographic information; 3D view; checkCIF report

## Figures and Tables

**Table 1:** Selected geometric parameters (Å, °)

N1—C5	1.357 (4)	N1′—C5′	1.343 (4)
N1—N2	1.375 (3)	N1′—N2′	1.380 (3)
N2—C3	1.317 (4)	N2′—C3′	1.324 (4)
C3—C4	1.424 (4)	C3′—C4′	1.432 (4)
C4—C5	1.392 (4)	C4′—C5′	1.390 (4)
C12—O1	1.221 (3)	C12′—O1′	1.228 (3)
C13—N3	1.145 (4)	C13′—N3′	1.155 (4)
C23—Br1	1.911 (3)	C23′—Br1′	1.906 (3)
			
C5—N1—N2	112.2 (2)	C5′—N1′—N2′	112.5 (2)
C5—N1—C14	128.3 (2)	C5′—N1′—C14′	128.5 (2)
C3—N2—N1	104.9 (2)	C3′—N2′—N1′	104.6 (2)
N2—C3—C4	111.9 (2)	N2′—C3′—C4′	111.5 (3)
C4—C3—C6	128.0 (3)	C4′—C3′—C6′	128.2 (3)
C5—C4—C3	104.5 (2)	C5′—C4′—C10′	131.0 (3)
C5—C4—C10	130.7 (3)	C5′—C4′—C3′	104.3 (2)
N1—C5—C4	106.5 (2)	N1′—C5′—C4′	107.0 (2)
C11—C10—C4	130.1 (3)	C11′—C10′—C4′	130.4 (3)
			
N2—C3—C6—C7	−107.2 (3)	N2′—C3′—C6′—C7′	−102.1 (3)
C4—C3—C6—C7	73.5 (4)	C4′—C3′—C6′—C7′	78.5 (4)
C10—C11—C12—N4	156.4 (3)	C10′—C11′—C12′—N4′	155.5 (3)
C5—N1—C14—C15	10.7 (5)	C5′—N1′—C14′—C15′	23.4 (5)
N2—N1—C14—C15	−173.4 (3)	N2′—N1′—C14′—C15′	−159.4 (3)

**Table 2:** Hydrogen-bond geometry (Å, °)

*D*—H⋯*A*	*D*—H	H⋯*A*	*D*⋯*A*	*D*—H⋯*A*
N4—H4⋯N3′	0.82 (3)	2.31 (3)	3.107 (3)	162 (3)
N4′—H4′⋯N3	0.82 (3)	2.32 (4)	3.097 (3)	158 (4)
C15—H15⋯O1′^i^	0.95	2.50	3.417 (4)	162
C15′—H15′⋯O1^ii^	0.95	2.51	3.455 (4)	174
C16′—H16′⋯Br1^iii^	0.95	2.91	3.813 (3)	159
C16—H16⋯Br1′^iii^	0.95	2.89	3.726 (3)	148

Symmetry codes: (i) 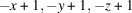 ; (ii) 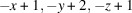 ; (iii) 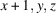 .

**Table 3:** Experimental details

Crystal data	
Chemical formula	C_23_H_21_BrN_4_O
*M*_r_	449.35
Crystal system, space group	Triclinic, *P* 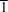
Temperature (K)	100
*a*, *b*, *c* (Å)	9.2260 (6), 12.7703 (9), 18.6607 (13)
α, β, γ (°)	109.790 (6), 96.720 (6), 90.657 (6)
*V* (Å^3^)	2051.4 (3)
*Z*	4
Radiation type	Cu *K*α
μ (mm^−1^)	2.90
Crystal size (mm)	0.15 × 0.08 × 0.02

Data collection	
Diffractometer	XtaLAB Synergy
Absorption correction	Multi-scan (*CrysAlis PRO*; Rigaku OD, 2023 ▸)
*T*_min_, *T*_max_	0.778, 1.000
No. of measured, independent and observed [*I* > 2σ(*I*)] reflections	8658, 8658, 8436
θ values (°)	θ_max_ = 80.7, θ_min_ = 2.5
(sin θ/λ)_max_ (Å^−1^)	0.640

Refinement	
*R*[*F* ^2^ > 2σ(*F* ^2^)], *wR*(*F* ^2^), *S*	0.040, 0.102, 1.05
No. of reflections	8658
No. of parameters	536
No. of restraints	1
H-atom treatment	H atoms treated by a mixture of independent and constrained refinement
Δρ_max_, Δρ_min_ (e Å^−3^)	1.45, −0.75

Computer programs: *CrysAlis PRO* (Rigaku OD, 2023 ▸), *SHELXT* (Sheldrick, 2015*a* ▸), *SHELXL2019/3* (Sheldrick, 2015*b* ▸) and *XP* (Bruker, 1998 ▸).

## supplementary crystallographic information

### Crystal data

**Table:**

C_23_H_21_BrN_4_O	*Z* = 4
*M**_r_* = 449.35	*F*(000) = 920
Triclinic, *P*1	*D*_x_ = 1.455 Mg m^−^^3^
*a* = 9.2260 (6) Å	Cu *K*α radiation, λ = 1.54184 Å
*b* = 12.7703 (9) Å	Cell parameters from 28092 reflections
*c* = 18.6607 (13) Å	θ = 4.8–80.0°
α = 109.790 (6)°	µ = 2.90 mm^−^^1^
β = 96.720 (6)°	*T* = 100 K
γ = 90.657 (6)°	Lath, colourless
*V* = 2051.4 (3) Å^3^	0.15 × 0.08 × 0.02 mm

### Data collection

**Table:**

XtaLAB Synergy diffractometer	8658 measured reflections
Radiation source: micro-focus sealed X-ray tube, PhotonJet (Cu) X-ray Source	8658 independent reflections
Mirror monochromator	8436 reflections with *I* > 2σ(*I*)
Detector resolution: 10.0000 pixels mm^-1^	θ_max_ = 80.7°, θ_min_ = 2.5°
ω scans	*h* = −11→11
Absorption correction: multi-scan (CrysAlisPro; Rigaku OD, 2023)	*k* = −16→16
*T*_min_ = 0.778, *T*_max_ = 1.000	*l* = −23→23

### Refinement

**Table:**

Refinement on *F*^2^	Primary atom site location: dual
Least-squares matrix: full	Hydrogen site location: mixed
*R*[*F*^2^ > 2σ(*F*^2^)] = 0.040	H atoms treated by a mixture of independent and constrained refinement
*wR*(*F*^2^) = 0.102	*w* = 1/[σ^2^(*F*_o_^2^) + (0.0504*P*)^2^ + 3.4902*P*] where *P* = (*F*_o_^2^ + 2*F*_c_^2^)/3
*S* = 1.05	(Δ/σ)_max_ = 0.002
8658 reflections	Δρ_max_ = 1.45 e Å^−^^3^
536 parameters	Δρ_min_ = −0.75 e Å^−^^3^
1 restraint	

### Fractional atomic coordinates and isotropic or equivalent isotropic displacement parameters (Å^2^)

**Table:**

	*x*	*y*	*z*	*U*_iso_*/*U*_eq_	
N1	0.8296 (3)	0.93208 (19)	0.80174 (13)	0.0195 (5)	
N2	0.8728 (3)	1.0434 (2)	0.83283 (14)	0.0223 (5)	
C3	0.7898 (3)	1.0913 (2)	0.79189 (16)	0.0204 (5)	
C4	0.6899 (3)	1.0129 (2)	0.73414 (15)	0.0192 (5)	
C5	0.7208 (3)	0.9108 (2)	0.74228 (15)	0.0194 (5)	
H5	0.674853	0.840171	0.712139	0.023*	
C6	0.8085 (3)	1.2144 (2)	0.80848 (17)	0.0234 (6)	
H6A	0.814865	1.227362	0.759512	0.028*	
H6B	0.902540	1.242139	0.841653	0.028*	
C7	0.6856 (4)	1.2837 (3)	0.84792 (18)	0.0269 (6)	
H7	0.592945	1.261373	0.811598	0.032*	
C8	0.6612 (5)	1.2636 (3)	0.9211 (2)	0.0364 (8)	
H8A	0.633489	1.184622	0.909325	0.055*	
H8B	0.582845	1.309233	0.944196	0.055*	
H8C	0.751428	1.283761	0.957285	0.055*	
C9	0.7220 (4)	1.4066 (3)	0.8637 (2)	0.0331 (7)	
H9A	0.814866	1.429449	0.897552	0.050*	
H9B	0.644192	1.450826	0.888617	0.050*	
H9C	0.730456	1.418601	0.815190	0.050*	
C10	0.5809 (3)	1.0408 (2)	0.68333 (15)	0.0188 (5)	
H10	0.581156	1.117548	0.688873	0.023*	
C11	0.4770 (3)	0.9745 (2)	0.62836 (16)	0.0191 (5)	
C12	0.3728 (3)	1.0298 (2)	0.58637 (15)	0.0188 (5)	
C13	0.4618 (3)	0.8564 (2)	0.60891 (16)	0.0215 (6)	
N3	0.4422 (3)	0.7617 (2)	0.59075 (15)	0.0274 (5)	
N4	0.3098 (3)	0.9632 (2)	0.51592 (14)	0.0204 (5)	
H4	0.334 (4)	0.899 (3)	0.497 (2)	0.026 (9)*	
O1	0.3486 (2)	1.12794 (16)	0.61581 (11)	0.0224 (4)	
C14	0.8933 (3)	0.8565 (2)	0.83581 (16)	0.0217 (6)	
C15	0.8624 (3)	0.7422 (2)	0.80079 (17)	0.0242 (6)	
H15	0.801738	0.713321	0.752930	0.029*	
C16	0.9223 (4)	0.6715 (3)	0.83729 (18)	0.0279 (6)	
H16	0.901761	0.593388	0.814056	0.033*	
C17	1.0113 (4)	0.7126 (3)	0.90700 (18)	0.0282 (6)	
H17	1.050090	0.663052	0.931612	0.034*	
C18	1.0436 (4)	0.8263 (3)	0.94063 (18)	0.0301 (7)	
H18	1.105799	0.854788	0.988046	0.036*	
C19	0.9854 (4)	0.8986 (3)	0.90518 (18)	0.0278 (6)	
H19	1.008014	0.976494	0.928011	0.033*	
C20	0.2082 (3)	0.9978 (2)	0.46651 (15)	0.0181 (5)	
C21	0.2181 (3)	1.1050 (2)	0.46342 (16)	0.0197 (5)	
H21	0.294543	1.156414	0.494685	0.024*	
C22	0.1169 (3)	1.1369 (2)	0.41490 (16)	0.0211 (5)	
H22	0.122820	1.209905	0.412529	0.025*	
C23	0.0071 (3)	1.0600 (2)	0.36994 (16)	0.0205 (5)	
Br1	−0.13165 (3)	1.10741 (2)	0.30445 (2)	0.02507 (8)	
C24	−0.0026 (3)	0.9522 (2)	0.37003 (17)	0.0227 (6)	
H24	−0.076916	0.900263	0.337075	0.027*	
C25	0.0985 (3)	0.9211 (2)	0.41927 (16)	0.0220 (6)	
H25	0.092915	0.847572	0.420802	0.026*	
N1'	0.6634 (3)	0.5581 (2)	0.19407 (13)	0.0212 (5)	
N2'	0.6975 (3)	0.4479 (2)	0.16639 (14)	0.0232 (5)	
C3'	0.6367 (3)	0.4009 (2)	0.20969 (16)	0.0219 (6)	
C4'	0.5604 (3)	0.4795 (2)	0.26490 (16)	0.0206 (5)	
C5'	0.5837 (3)	0.5796 (2)	0.25271 (16)	0.0204 (5)	
H5'	0.549638	0.649800	0.280581	0.024*	
C6'	0.6537 (3)	0.2800 (2)	0.19788 (18)	0.0237 (6)	
H6A'	0.675875	0.271529	0.248784	0.028*	
H6B'	0.738793	0.255432	0.169796	0.028*	
C7'	0.5204 (4)	0.2024 (3)	0.15374 (18)	0.0290 (7)	
H7'	0.437221	0.223875	0.184710	0.035*	
C8'	0.4739 (6)	0.2124 (4)	0.0759 (2)	0.0559 (13)	
H8A'	0.448415	0.289057	0.082456	0.067*	
H8B'	0.388753	0.161378	0.050055	0.067*	
H8C'	0.554472	0.193070	0.044653	0.067*	
C9'	0.5519 (4)	0.0823 (3)	0.1454 (2)	0.0364 (8)	
H9A'	0.579375	0.076961	0.196370	0.044*	
H9B'	0.632326	0.058781	0.114547	0.044*	
H9C'	0.464345	0.033882	0.120015	0.044*	
C10'	0.4783 (3)	0.4527 (2)	0.31708 (16)	0.0197 (5)	
H10'	0.475359	0.376301	0.312449	0.024*	
C11'	0.4033 (3)	0.5191 (2)	0.37249 (16)	0.0190 (5)	
C12'	0.3216 (3)	0.4648 (2)	0.41656 (16)	0.0201 (5)	
C13'	0.3989 (3)	0.6369 (2)	0.39099 (16)	0.0209 (5)	
N3'	0.3903 (3)	0.7323 (2)	0.40865 (15)	0.0274 (5)	
N4'	0.2974 (3)	0.5319 (2)	0.48735 (14)	0.0206 (5)	
H4'	0.328 (5)	0.597 (3)	0.504 (3)	0.046 (13)*	
O1'	0.2807 (2)	0.36590 (16)	0.38837 (11)	0.0221 (4)	
C14'	0.7091 (3)	0.6320 (2)	0.15749 (17)	0.0227 (6)	
C15'	0.7193 (3)	0.7463 (2)	0.19677 (17)	0.0234 (6)	
H15'	0.696881	0.775749	0.247998	0.028*	
C16'	0.7630 (4)	0.8168 (3)	0.15950 (18)	0.0276 (6)	
H16'	0.770824	0.895077	0.185682	0.033*	
C17'	0.7951 (4)	0.7743 (3)	0.08488 (19)	0.0288 (6)	
H17'	0.824273	0.823276	0.059927	0.035*	
C18'	0.7847 (4)	0.6603 (3)	0.04658 (19)	0.0322 (7)	
H18'	0.807749	0.631370	−0.004554	0.039*	
C19'	0.7409 (4)	0.5874 (3)	0.08222 (18)	0.0290 (7)	
H19'	0.732788	0.509238	0.055784	0.035*	
C20'	0.2234 (3)	0.4972 (2)	0.53829 (15)	0.0184 (5)	
C21'	0.2400 (3)	0.3921 (2)	0.54385 (16)	0.0199 (5)	
H21'	0.302404	0.342360	0.513082	0.024*	
C22'	0.1665 (3)	0.3593 (2)	0.59383 (16)	0.0216 (6)	
H22'	0.176915	0.287245	0.597366	0.026*	
C23'	0.0771 (3)	0.4338 (2)	0.63876 (16)	0.0207 (5)	
Br1'	−0.02465 (4)	0.38496 (3)	0.70597 (2)	0.02632 (8)	
C24'	0.0621 (3)	0.5396 (2)	0.63612 (17)	0.0237 (6)	
H24'	0.002619	0.589873	0.668537	0.028*	
C25'	0.1352 (3)	0.5716 (2)	0.58544 (17)	0.0221 (6)	
H25'	0.125442	0.644200	0.582714	0.026*	

### Atomic displacement parameters (Å^2^)

**Table:**

	*U*^11^	*U*^22^	*U*^33^	*U*^12^	*U*^13^	*U*^23^
N1	0.0240 (12)	0.0131 (11)	0.0200 (11)	0.0007 (9)	0.0023 (9)	0.0041 (8)
N2	0.0240 (12)	0.0152 (11)	0.0244 (12)	−0.0013 (9)	0.0012 (9)	0.0032 (9)
C3	0.0208 (14)	0.0169 (13)	0.0227 (13)	−0.0002 (10)	0.0034 (10)	0.0057 (10)
C4	0.0204 (13)	0.0170 (13)	0.0200 (12)	0.0001 (10)	0.0030 (10)	0.0060 (10)
C5	0.0225 (14)	0.0161 (12)	0.0181 (12)	0.0006 (10)	0.0005 (10)	0.0047 (10)
C6	0.0225 (14)	0.0172 (13)	0.0283 (14)	−0.0035 (11)	0.0002 (11)	0.0061 (11)
C7	0.0299 (16)	0.0214 (14)	0.0286 (15)	0.0015 (12)	0.0012 (12)	0.0082 (12)
C8	0.050 (2)	0.0303 (17)	0.0289 (16)	0.0076 (15)	0.0096 (15)	0.0092 (13)
C9	0.0348 (18)	0.0229 (15)	0.0378 (17)	0.0036 (13)	−0.0010 (14)	0.0073 (13)
C10	0.0205 (13)	0.0140 (12)	0.0218 (12)	0.0002 (10)	0.0045 (10)	0.0056 (10)
C11	0.0201 (13)	0.0172 (13)	0.0200 (12)	0.0027 (10)	0.0048 (10)	0.0058 (10)
C12	0.0190 (13)	0.0184 (13)	0.0190 (12)	−0.0003 (10)	0.0036 (10)	0.0059 (10)
C13	0.0204 (14)	0.0208 (14)	0.0222 (13)	0.0008 (11)	0.0008 (10)	0.0065 (11)
N3	0.0268 (13)	0.0190 (13)	0.0339 (13)	−0.0013 (10)	−0.0021 (11)	0.0080 (10)
N4	0.0239 (12)	0.0132 (11)	0.0218 (11)	0.0025 (9)	−0.0002 (9)	0.0037 (9)
O1	0.0269 (11)	0.0145 (9)	0.0224 (9)	0.0023 (8)	0.0007 (8)	0.0027 (7)
C14	0.0237 (14)	0.0203 (14)	0.0218 (13)	0.0027 (11)	0.0037 (11)	0.0077 (11)
C15	0.0266 (15)	0.0190 (14)	0.0247 (14)	0.0016 (11)	0.0029 (11)	0.0046 (11)
C16	0.0323 (17)	0.0207 (14)	0.0307 (15)	0.0062 (12)	0.0060 (13)	0.0080 (12)
C17	0.0335 (17)	0.0255 (15)	0.0304 (15)	0.0091 (13)	0.0076 (13)	0.0141 (12)
C18	0.0335 (17)	0.0307 (16)	0.0246 (14)	0.0057 (13)	−0.0021 (12)	0.0092 (12)
C19	0.0339 (17)	0.0206 (14)	0.0266 (14)	0.0029 (12)	−0.0017 (12)	0.0067 (11)
C20	0.0187 (13)	0.0143 (12)	0.0205 (12)	0.0035 (10)	0.0027 (10)	0.0050 (10)
C21	0.0199 (13)	0.0158 (12)	0.0204 (12)	−0.0012 (10)	0.0030 (10)	0.0022 (10)
C22	0.0258 (15)	0.0155 (12)	0.0210 (13)	0.0007 (11)	0.0026 (11)	0.0052 (10)
C23	0.0198 (13)	0.0213 (13)	0.0187 (12)	0.0043 (11)	0.0028 (10)	0.0044 (10)
Br1	0.02476 (15)	0.02211 (15)	0.02525 (15)	0.00458 (11)	−0.00201 (11)	0.00573 (12)
C24	0.0220 (14)	0.0188 (13)	0.0247 (14)	−0.0009 (11)	0.0003 (11)	0.0050 (11)
C25	0.0257 (14)	0.0148 (12)	0.0237 (13)	0.0000 (11)	0.0036 (11)	0.0041 (10)
N1'	0.0272 (13)	0.0144 (11)	0.0218 (11)	0.0036 (9)	0.0049 (9)	0.0054 (9)
N2'	0.0256 (13)	0.0155 (11)	0.0261 (12)	0.0017 (9)	0.0046 (10)	0.0034 (9)
C3'	0.0232 (14)	0.0191 (13)	0.0219 (13)	0.0019 (11)	0.0030 (11)	0.0050 (11)
C4'	0.0207 (14)	0.0189 (13)	0.0210 (13)	0.0035 (10)	0.0017 (10)	0.0054 (10)
C5'	0.0218 (14)	0.0184 (13)	0.0206 (12)	0.0054 (10)	0.0056 (10)	0.0051 (10)
C6'	0.0260 (15)	0.0175 (13)	0.0291 (14)	0.0047 (11)	0.0072 (11)	0.0089 (11)
C7'	0.0369 (18)	0.0221 (15)	0.0282 (15)	0.0012 (13)	0.0033 (13)	0.0091 (12)
C8'	0.089 (4)	0.038 (2)	0.035 (2)	−0.019 (2)	−0.018 (2)	0.0147 (17)
C9'	0.043 (2)	0.0219 (16)	0.0423 (19)	−0.0015 (14)	0.0102 (15)	0.0075 (14)
C10'	0.0214 (13)	0.0161 (12)	0.0207 (12)	0.0008 (10)	0.0011 (10)	0.0057 (10)
C11'	0.0185 (13)	0.0154 (12)	0.0219 (13)	0.0010 (10)	−0.0002 (10)	0.0059 (10)
C12'	0.0196 (13)	0.0183 (13)	0.0212 (13)	0.0018 (10)	0.0017 (10)	0.0056 (10)
C13'	0.0212 (14)	0.0196 (14)	0.0223 (13)	0.0015 (11)	0.0037 (10)	0.0073 (11)
N3'	0.0300 (14)	0.0213 (13)	0.0323 (13)	0.0024 (10)	0.0089 (11)	0.0093 (10)
N4'	0.0250 (12)	0.0136 (11)	0.0221 (11)	−0.0015 (9)	0.0054 (9)	0.0040 (9)
O1'	0.0274 (11)	0.0142 (9)	0.0223 (9)	−0.0023 (8)	0.0034 (8)	0.0032 (7)
C14'	0.0242 (14)	0.0211 (14)	0.0240 (13)	−0.0011 (11)	0.0044 (11)	0.0090 (11)
C15'	0.0274 (15)	0.0198 (14)	0.0221 (13)	−0.0014 (11)	0.0018 (11)	0.0067 (11)
C16'	0.0309 (16)	0.0209 (14)	0.0295 (15)	−0.0058 (12)	−0.0014 (12)	0.0087 (12)
C17'	0.0325 (17)	0.0258 (15)	0.0300 (15)	−0.0031 (13)	0.0011 (13)	0.0132 (12)
C18'	0.046 (2)	0.0257 (16)	0.0248 (15)	−0.0039 (14)	0.0083 (13)	0.0072 (12)
C19'	0.0404 (18)	0.0198 (14)	0.0265 (14)	−0.0001 (13)	0.0082 (13)	0.0061 (12)
C20'	0.0194 (13)	0.0152 (12)	0.0195 (12)	−0.0008 (10)	0.0014 (10)	0.0048 (10)
C21'	0.0207 (13)	0.0148 (12)	0.0213 (13)	0.0023 (10)	0.0029 (10)	0.0021 (10)
C22'	0.0268 (15)	0.0161 (13)	0.0207 (13)	0.0011 (11)	0.0023 (11)	0.0050 (10)
C23'	0.0211 (14)	0.0203 (13)	0.0202 (13)	−0.0030 (11)	0.0018 (10)	0.0066 (10)
Br1'	0.02965 (17)	0.02193 (15)	0.02696 (15)	−0.00282 (11)	0.00926 (12)	0.00613 (12)
C24'	0.0242 (15)	0.0217 (14)	0.0235 (13)	0.0035 (11)	0.0059 (11)	0.0048 (11)
C25'	0.0255 (14)	0.0134 (12)	0.0268 (14)	0.0033 (11)	0.0029 (11)	0.0063 (10)

### Geometric parameters (Å, º)

**Table:**

N1—C5	1.357 (4)	C16'—C17'	1.382 (5)
N1—N2	1.375 (3)	C17'—C18'	1.384 (5)
N1—C14	1.423 (4)	C18'—C19'	1.395 (4)
N2—C3	1.317 (4)	C20'—C21'	1.390 (4)
C3—C4	1.424 (4)	C20'—C25'	1.399 (4)
C3—C6	1.499 (4)	C21'—C22'	1.382 (4)
C4—C5	1.392 (4)	C22'—C23'	1.388 (4)
C4—C10	1.432 (4)	C23'—C24'	1.377 (4)
C6—C7	1.545 (4)	C23'—Br1'	1.906 (3)
C7—C8	1.514 (5)	C24'—C25'	1.386 (4)
C7—C9	1.521 (4)	C5—H5	0.9500
C10—C11	1.361 (4)	C6—H6A	0.9900
C11—C13	1.428 (4)	C6—H6B	0.9900
C11—C12	1.500 (4)	C7—H7	1.0000
C12—O1	1.221 (3)	C8—H8A	0.9800
C12—N4	1.357 (4)	C8—H8B	0.9800
C13—N3	1.145 (4)	C8—H8C	0.9800
N4—C20	1.417 (4)	C9—H9A	0.9800
C14—C15	1.392 (4)	C9—H9B	0.9800
C14—C19	1.395 (4)	C9—H9C	0.9800
C15—C16	1.387 (4)	C10—H10	0.9500
C16—C17	1.386 (5)	N4—H4	0.82 (3)
C17—C18	1.386 (5)	C15—H15	0.9500
C18—C19	1.385 (4)	C16—H16	0.9500
C20—C21	1.391 (4)	C17—H17	0.9500
C20—C25	1.395 (4)	C18—H18	0.9500
C21—C22	1.385 (4)	C19—H19	0.9500
C22—C23	1.384 (4)	C21—H21	0.9500
C23—C24	1.379 (4)	C22—H22	0.9500
C23—Br1	1.911 (3)	C24—H24	0.9500
C24—C25	1.388 (4)	C25—H25	0.9500
N1'—C5'	1.343 (4)	C5'—H5'	0.9500
N1'—N2'	1.380 (3)	C6'—H6A'	0.9900
N1'—C14'	1.425 (4)	C6'—H6B'	0.9900
N2'—C3'	1.324 (4)	C7'—H7'	1.0000
C3'—C4'	1.432 (4)	C8'—H8A'	0.9800
C3'—C6'	1.497 (4)	C8'—H8B'	0.9800
C4'—C5'	1.390 (4)	C8'—H8C'	0.9800
C4'—C10'	1.425 (4)	C9'—H9A'	0.9800
C6'—C7'	1.534 (4)	C9'—H9B'	0.9800
C7'—C8'	1.514 (5)	C9'—H9C'	0.9800
C7'—C9'	1.523 (5)	C10'—H10'	0.9500
C10'—C11'	1.361 (4)	N4'—H4'	0.82 (3)
C11'—C13'	1.427 (4)	C15'—H15'	0.9500
C11'—C12'	1.498 (4)	C16'—H16'	0.9500
C12'—O1'	1.228 (3)	C17'—H17'	0.9500
C12'—N4'	1.355 (4)	C18'—H18'	0.9500
C13'—N3'	1.155 (4)	C19'—H19'	0.9500
N4'—C20'	1.413 (4)	C21'—H21'	0.9500
C14'—C15'	1.390 (4)	C22'—H22'	0.9500
C14'—C19'	1.393 (4)	C24'—H24'	0.9500
C15'—C16'	1.392 (4)	C25'—H25'	0.9500
			
C5—N1—N2	112.2 (2)	C3—C6—H6A	108.5
C5—N1—C14	128.3 (2)	C7—C6—H6A	108.5
N2—N1—C14	119.4 (2)	C3—C6—H6B	108.5
C3—N2—N1	104.9 (2)	C7—C6—H6B	108.5
N2—C3—C4	111.9 (2)	H6A—C6—H6B	107.5
N2—C3—C6	120.1 (3)	C8—C7—H7	108.1
C4—C3—C6	128.0 (3)	C9—C7—H7	108.1
C5—C4—C3	104.5 (2)	C6—C7—H7	108.1
C5—C4—C10	130.7 (3)	C7—C8—H8A	109.5
C3—C4—C10	124.8 (3)	C7—C8—H8B	109.5
N1—C5—C4	106.5 (2)	H8A—C8—H8B	109.5
C3—C6—C7	115.1 (3)	C7—C8—H8C	109.5
C8—C7—C9	110.8 (3)	H8A—C8—H8C	109.5
C8—C7—C6	112.3 (3)	H8B—C8—H8C	109.5
C9—C7—C6	109.2 (3)	C7—C9—H9A	109.5
C11—C10—C4	130.1 (3)	C7—C9—H9B	109.5
C10—C11—C13	123.8 (3)	H9A—C9—H9B	109.5
C10—C11—C12	117.5 (2)	C7—C9—H9C	109.5
C13—C11—C12	118.7 (2)	H9A—C9—H9C	109.5
O1—C12—N4	124.3 (3)	H9B—C9—H9C	109.5
O1—C12—C11	120.4 (2)	C11—C10—H10	115.0
N4—C12—C11	115.3 (2)	C4—C10—H10	115.0
N3—C13—C11	176.0 (3)	C12—N4—H4	121 (3)
C12—N4—C20	124.9 (2)	C20—N4—H4	114 (3)
C15—C14—C19	120.6 (3)	C16—C15—H15	120.7
C15—C14—N1	120.3 (3)	C14—C15—H15	120.7
C19—C14—N1	119.0 (3)	C17—C16—H16	119.4
C16—C15—C14	118.5 (3)	C15—C16—H16	119.4
C17—C16—C15	121.3 (3)	C16—C17—H17	120.2
C16—C17—C18	119.7 (3)	C18—C17—H17	120.2
C19—C18—C17	120.1 (3)	C19—C18—H18	120.0
C18—C19—C14	119.7 (3)	C17—C18—H18	120.0
C21—C20—C25	120.1 (3)	C18—C19—H19	120.1
C21—C20—N4	121.4 (2)	C14—C19—H19	120.1
C25—C20—N4	118.5 (2)	C22—C21—H21	119.9
C22—C21—C20	120.2 (3)	C20—C21—H21	119.9
C23—C22—C21	118.6 (3)	C23—C22—H22	120.7
C24—C23—C22	122.5 (3)	C21—C22—H22	120.7
C24—C23—Br1	120.1 (2)	C23—C24—H24	120.7
C22—C23—Br1	117.4 (2)	C25—C24—H24	120.7
C23—C24—C25	118.6 (3)	C24—C25—H25	120.0
C24—C25—C20	120.0 (3)	C20—C25—H25	120.0
C5'—N1'—N2'	112.5 (2)	N1'—C5'—H5'	126.5
C5'—N1'—C14'	128.5 (2)	C4'—C5'—H5'	126.5
N2'—N1'—C14'	118.9 (2)	C3'—C6'—H6A'	108.5
C3'—N2'—N1'	104.6 (2)	C7'—C6'—H6A'	108.5
N2'—C3'—C4'	111.5 (3)	C3'—C6'—H6B'	108.5
N2'—C3'—C6'	120.3 (3)	C7'—C6'—H6B'	108.5
C4'—C3'—C6'	128.2 (3)	H6A'—C6'—H6B'	107.5
C5'—C4'—C10'	131.0 (3)	C8'—C7'—H7'	107.9
C5'—C4'—C3'	104.3 (2)	C9'—C7'—H7'	107.9
C10'—C4'—C3'	124.6 (3)	C6'—C7'—H7'	107.9
N1'—C5'—C4'	107.0 (2)	C7'—C8'—H8A'	109.5
C3'—C6'—C7'	115.2 (3)	C7'—C8'—H8B'	109.5
C8'—C7'—C9'	110.5 (3)	H8A'—C8'—H8B'	109.5
C8'—C7'—C6'	112.6 (3)	C7'—C8'—H8C'	109.5
C9'—C7'—C6'	110.0 (3)	H8A'—C8'—H8C'	109.5
C11'—C10'—C4'	130.4 (3)	H8B'—C8'—H8C'	109.5
C10'—C11'—C13'	123.8 (3)	C7'—C9'—H9A'	109.5
C10'—C11'—C12'	117.7 (2)	C7'—C9'—H9B'	109.5
C13'—C11'—C12'	118.5 (2)	H9A'—C9'—H9B'	109.5
O1'—C12'—N4'	124.0 (3)	C7'—C9'—H9C'	109.5
O1'—C12'—C11'	120.5 (3)	H9A'—C9'—H9C'	109.5
N4'—C12'—C11'	115.5 (2)	H9B'—C9'—H9C'	109.5
N3'—C13'—C11'	176.4 (3)	C11'—C10'—H10'	114.8
C12'—N4'—C20'	124.6 (2)	C4'—C10'—H10'	114.8
C15'—C14'—C19'	121.5 (3)	C12'—N4'—H4'	120 (3)
C15'—C14'—N1'	119.8 (3)	C20'—N4'—H4'	115 (3)
C19'—C14'—N1'	118.7 (3)	C14'—C15'—H15'	120.7
C14'—C15'—C16'	118.6 (3)	C16'—C15'—H15'	120.7
C17'—C16'—C15'	120.8 (3)	C17'—C16'—H16'	119.6
C16'—C17'—C18'	119.9 (3)	C15'—C16'—H16'	119.6
C17'—C18'—C19'	120.7 (3)	C16'—C17'—H17'	120.1
C14'—C19'—C18'	118.5 (3)	C18'—C17'—H17'	120.1
C21'—C20'—C25'	119.7 (3)	C17'—C18'—H18'	119.6
C21'—C20'—N4'	121.2 (2)	C19'—C18'—H18'	119.6
C25'—C20'—N4'	119.1 (2)	C14'—C19'—H19'	120.8
C22'—C21'—C20'	120.5 (3)	C18'—C19'—H19'	120.8
C21'—C22'—C23'	118.7 (3)	C22'—C21'—H21'	119.8
C24'—C23'—C22'	122.0 (3)	C20'—C21'—H21'	119.8
C24'—C23'—Br1'	120.4 (2)	C21'—C22'—H22'	120.6
C22'—C23'—Br1'	117.5 (2)	C23'—C22'—H22'	120.6
C23'—C24'—C25'	119.0 (3)	C23'—C24'—H24'	120.5
C24'—C25'—C20'	120.1 (3)	C25'—C24'—H24'	120.5
N1—C5—H5	126.7	C24'—C25'—H25'	120.0
C4—C5—H5	126.7	C20'—C25'—H25'	120.0
			
C5—N1—N2—C3	−0.1 (3)	C5'—N1'—N2'—C3'	−0.1 (3)
C14—N1—N2—C3	−176.7 (2)	C14'—N1'—N2'—C3'	−177.8 (3)
N1—N2—C3—C4	0.7 (3)	N1'—N2'—C3'—C4'	1.1 (3)
N1—N2—C3—C6	−178.7 (2)	N1'—N2'—C3'—C6'	−178.4 (3)
N2—C3—C4—C5	−1.0 (3)	N2'—C3'—C4'—C5'	−1.6 (3)
C6—C3—C4—C5	178.3 (3)	C6'—C3'—C4'—C5'	177.8 (3)
N2—C3—C4—C10	176.7 (3)	N2'—C3'—C4'—C10'	176.9 (3)
C6—C3—C4—C10	−4.0 (5)	C6'—C3'—C4'—C10'	−3.7 (5)
N2—N1—C5—C4	−0.5 (3)	N2'—N1'—C5'—C4'	−0.9 (3)
C14—N1—C5—C4	175.6 (3)	C14'—N1'—C5'—C4'	176.5 (3)
C3—C4—C5—N1	0.9 (3)	C10'—C4'—C5'—N1'	−176.9 (3)
C10—C4—C5—N1	−176.6 (3)	C3'—C4'—C5'—N1'	1.4 (3)
N2—C3—C6—C7	−107.2 (3)	N2'—C3'—C6'—C7'	−102.1 (3)
C4—C3—C6—C7	73.5 (4)	C4'—C3'—C6'—C7'	78.5 (4)
C3—C6—C7—C8	54.6 (4)	C3'—C6'—C7'—C8'	56.1 (4)
C3—C6—C7—C9	177.9 (3)	C3'—C6'—C7'—C9'	179.8 (3)
C5—C4—C10—C11	−0.3 (5)	C5'—C4'—C10'—C11'	−3.0 (5)
C3—C4—C10—C11	−177.4 (3)	C3'—C4'—C10'—C11'	178.9 (3)
C4—C10—C11—C13	−2.5 (5)	C4'—C10'—C11'—C13'	−2.7 (5)
C4—C10—C11—C12	177.3 (3)	C4'—C10'—C11'—C12'	177.6 (3)
C10—C11—C12—O1	−24.1 (4)	C10'—C11'—C12'—O1'	−24.7 (4)
C13—C11—C12—O1	155.7 (3)	C13'—C11'—C12'—O1'	155.5 (3)
C10—C11—C12—N4	156.4 (3)	C10'—C11'—C12'—N4'	155.5 (3)
C13—C11—C12—N4	−23.8 (4)	C13'—C11'—C12'—N4'	−24.2 (4)
O1—C12—N4—C20	0.4 (5)	O1'—C12'—N4'—C20'	0.6 (5)
C11—C12—N4—C20	179.9 (3)	C11'—C12'—N4'—C20'	−179.6 (3)
C5—N1—C14—C15	10.7 (5)	C5'—N1'—C14'—C15'	23.4 (5)
N2—N1—C14—C15	−173.4 (3)	N2'—N1'—C14'—C15'	−159.4 (3)
C5—N1—C14—C19	−168.4 (3)	C5'—N1'—C14'—C19'	−155.8 (3)
N2—N1—C14—C19	7.5 (4)	N2'—N1'—C14'—C19'	21.5 (4)
C19—C14—C15—C16	1.6 (5)	C19'—C14'—C15'—C16'	−0.3 (5)
N1—C14—C15—C16	−177.6 (3)	N1'—C14'—C15'—C16'	−179.5 (3)
C14—C15—C16—C17	−0.2 (5)	C14'—C15'—C16'—C17'	0.3 (5)
C15—C16—C17—C18	−1.0 (5)	C15'—C16'—C17'—C18'	−0.4 (5)
C16—C17—C18—C19	0.9 (5)	C16'—C17'—C18'—C19'	0.6 (5)
C17—C18—C19—C14	0.4 (5)	C15'—C14'—C19'—C18'	0.5 (5)
C15—C14—C19—C18	−1.7 (5)	N1'—C14'—C19'—C18'	179.6 (3)
N1—C14—C19—C18	177.4 (3)	C17'—C18'—C19'—C14'	−0.6 (5)
C12—N4—C20—C21	34.5 (4)	C12'—N4'—C20'—C21'	37.1 (4)
C12—N4—C20—C25	−146.8 (3)	C12'—N4'—C20'—C25'	−144.8 (3)
C25—C20—C21—C22	1.7 (4)	C25'—C20'—C21'—C22'	2.1 (4)
N4—C20—C21—C22	−179.7 (3)	N4'—C20'—C21'—C22'	−179.8 (3)
C20—C21—C22—C23	−0.1 (4)	C20'—C21'—C22'—C23'	−0.6 (4)
C21—C22—C23—C24	−2.0 (4)	C21'—C22'—C23'—C24'	−1.4 (4)
C21—C22—C23—Br1	179.4 (2)	C21'—C22'—C23'—Br1'	179.1 (2)
C22—C23—C24—C25	2.5 (4)	C22'—C23'—C24'—C25'	1.9 (4)
Br1—C23—C24—C25	−178.9 (2)	Br1'—C23'—C24'—C25'	−178.6 (2)
C23—C24—C25—C20	−0.9 (4)	C23'—C24'—C25'—C20'	−0.4 (4)
C21—C20—C25—C24	−1.1 (4)	C21'—C20'—C25'—C24'	−1.5 (4)
N4—C20—C25—C24	−179.8 (3)	N4'—C20'—C25'—C24'	−179.7 (3)

### Hydrogen-bond geometry (Å, º)

**Table:**

*D*—H···*A*	*D*—H	H···*A*	*D*···*A*	*D*—H···*A*
N4—H4···N3′	0.82 (3)	2.31 (3)	3.107 (3)	162 (3)
N4′—H4′···N3	0.82 (3)	2.32 (4)	3.097 (3)	158 (4)
C15—H15···O1′^i^	0.95	2.50	3.417 (4)	162
C15′—H15′···O1^ii^	0.95	2.51	3.455 (4)	174
C16′—H16′···Br1^iii^	0.95	2.91	3.813 (3)	159
C16—H16···Br1′^iii^	0.95	2.89	3.726 (3)	148

Symmetry codes: (i) −*x*+1, −*y*+1, −*z*+1; (ii) −*x*+1, −*y*+2, −*z*+1; (iii) *x*+1, *y*, *z*.
